# Fabrication and Characterization of Nanocarbon-Based Nanofluids by Using an Oxygen–Acetylene Flame Synthesis System

**DOI:** 10.1186/s11671-016-1522-6

**Published:** 2016-06-13

**Authors:** Tun-Ping Teng, Wei-Ping Wang, Yu-Chun Hsu

**Affiliations:** Department of Industrial Education, National Taiwan Normal University, No. 162, Section 1, He-ping East Road, Da-an District, Taipei, 10610 Taiwan, Republic of China

**Keywords:** Amorphous carbon (AC), Graphene oxide (GO), Nanocarbon-based nanofluid (NCBNF), Oxygen–acetylene flame synthesis system (OAFSS), Thermal conductivity

## Abstract

In this study, an oxygen–acetylene flame synthesis system was developed to fabricate nanocarbon-based nanofluids (NCBNFs) through a one-step synthesis method. Measured in liters per minute (LPM), the flame’s fuel flows combined oxygen and acetylene at four ratios: 1.5/2.5 (P1), 1.0/2.5 (P2), 0.5/2.5 (P3), and 0/2.5 (P4). The flow rate of cooling water (base fluid) was fixed at 1.2 LPM to produce different nanocarbon-based materials (NCBMs) and various concentrations of NCBNFs. Tests and analyses were conducted for determining the morphology of NCBMs, NCBM material, optical characteristics, the production rate, suspension performance, average particle size, zeta potential, and other relevant basic characteristics of NCBNFs to understand the characteristics and materials of NCBNFs produced through different process parameters (P1–P4). The results revealed that the NCBMs mainly had flaky and spherical morphologies and the diameters of the spherical NCBMs measured approximately 20–30 nm. X-ray diffraction and Raman spectroscopy revealed that the NCBMs contained graphene oxide (GO) and amorphous carbon (AC) when the oxygen flow rate was lower than 1.0 LPM. In addition, the NCBMs contained reduced GO, crystalline graphite (graphite-2H), and AC when the oxygen flow rate was higher than 1.0 LPM. The process parameters of P1, P2, P3, and P4 resulted in NCBMs produced at concentrations of 0.010, 0.013, 0.040, and 0.023 wt%, respectively, in NCBNFs. All the NCBNFs exhibited non-Newtonian and shear-thinning rheological properties. The P4 ratio showed the highest enhancement rate of thermal conductivity for NCBNFs, at a rate 4.85 % higher than that of water.

## Background

Nanofluids (NFs) are obtained by adding nanoparticles to conventional working fluids to form stable solid–liquid suspensions [1]. NFs can be used in many industries for improving system efficiency or for process improvements. Because NFs can be used to enhance the thermal properties of working fluids and the heat transfer efficiency of heat exchangers, many researchers have investigated NFs in depth, examining topics such as their manufacturing methods, basic characteristics (e.g., thermal conductivity, density, viscosity, specific heat, suspension capability), heat transfer behavior (for pipes and heat exchangers with different geometries), transport behavior (e.g., pressure drop, pumping power, and rheological properties), and the NFs used for improving the efficiencies of equipment such as heat dissipation radiators, heat recovery systems, and solar collectors [2–6].

In previous studies, nanoparticles (NPs) added to NFs have mostly been metal NPs (e.g., Cu, Ag, and Au) and oxide NPs (e.g., CuO, Al_2_O_3_, TiO_2_, SiO_2_, and ZnO). The thermal conductivity of metal NFs is typically higher than that of oxide NFs, but metal NPs in the base fluid are easily oxidized; therefore, metal NFs can soon be expected to be converted to oxide NFs. Furthermore, most metal NPs are expensive because mass production is difficult and they are not used in practical equipment. Although oxide NFs have characteristics that are fairly stable, their thermal conductivity is low and cannot be increased appreciably by raising the thermal conductivity of the base fluid. However, the high aspect ratio of NPs increases disturbance in the working fluid (microconvection), which can enhance the thermal conductivity and heat convective performance of NFs [7, 8].

Researchers have begun studying the manufacturing technology, characterization, and applications of carbon-based nanomaterials such as nanographites (NGs), nanocarbons (NCs), carbon nanotubes (CNTs), and graphene because of their high thermal conductivity [9–15], high heat transfer coefficient, heat exchange capacity in the base fluid [16–18], high aspect ratio, and unique mechanical and physicochemical properties [17–25]. Most of the thermal properties of CNTs and graphene are superior to those of NGs and NCs; therefore, NFs prepared by adding CNTs and graphene to the base fluid can be expected to exhibit excellent thermal performance. However, many methods used for manufacturing CNTs and graphene require a particular atmosphere or specific equipment, leading to high manufacturing costs or the use of numerous chemicals, which results in waste treatment problems. Therefore, when the use of CNTs and graphene is considered, associated requirements such as the manufacturing cost, the scale of production, and the environmental friendliness of the manufacturing process should also be considered.

This study employed the oxygen (O_2_)–acetylene (C_2_H_2_) flame synthesis method (OAFSM) to develop an O_2_–C_2_H_2_ flame synthesis system (OAFSS) for fabricating nanocarbon-based nanofluids (NCBNFs). This method was applied at four flow rate ratios of O_2_ to C_2_H_2_. The morphology, structure, particle size, suspension performance, and other basic characteristics of nanocarbon-based materials (NCBMs) and NCBNFs were tested using suitable instruments and test methods to demonstrate the characteristics of NCBMs and NCBNFs and the feasibility of manufacturing NCBNFs with this OAFSS.

## Methods

**Fig. 1:**
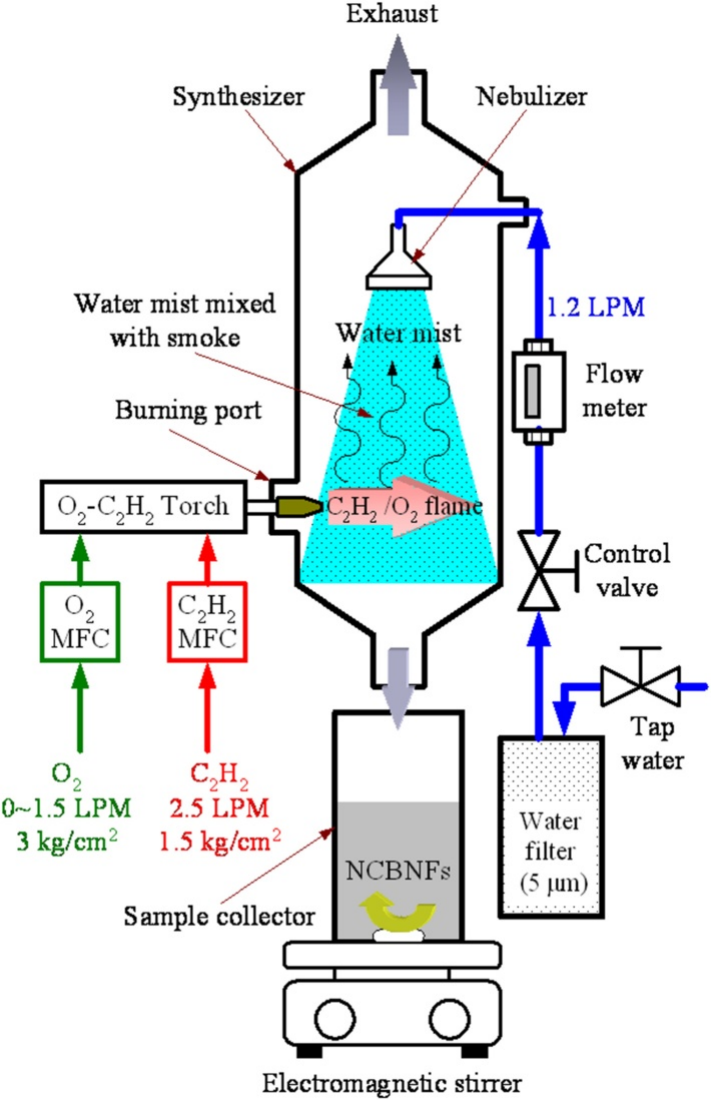
Schematic layout of the OAFSS for NCBNFs

Preparing the NCBNFs involved applying the OAFSM, which is a single-step synthesis method. Figure 1 displays a schematic of the OAFSS with the OAFM for NCBNFs. An O_2_–C_2_H_2_ flame was the carbon source. A nebulizer, synthesizer, sample collector, water flow meter, control valve, digital mass flow controller (MFC), electromagnetic stirrer (PC420D, Corning, USA), and O_2_–C_2_H_2_ torch were integrated to complete the OAFSS. As shown in Fig. 1, filtered water (tap water filtered and purified using a 5-μm filter) traversed a control valve and flow meter to control the spray state and flow rate of the nebulizer. The O_2_–C_2_H_2_ torch produced flames at different flow rates and ratios of O_2_–C_2_H_2_ that were controlled using the MFC. The O_2_–C_2_H_2_ flame was burned in a synthesizer as a carbon source; the generated smoke was cooled and condensed by water mist to form the NCBMs. When the mixtures of NCBMs and filtered water flowed into the sample collector, the mixtures were NCBNFs.

The manufacturing process parameters of the OAFSS for NCBNFs are detailed as follows. The flow rate of filtered water was controlled at 1.2 liters per minute (LPM), the C_2_H_2_ flow rate was fixed at 2.5 LPM at a pressure of 1.5 kg/cm^2^, and the O_2_ flow rate was controlled at 0–1.5 LPM at a pressure of 3.0 kg/cm^2^. The O_2_/C_2_H_2_ fuel combination had four configurations of the flow ratio, designated P1–P4 (P1 1.5/2.5, P2 1.0/2.5, P3 0.5/2.5, P4 0/2.5). An increase in the proportion of oxygen in the O_2_–C_2_H_2_ flame is expected to produce a more complete combustion, less smog and less NCBM, and vice versa. To reduce the risk of cross contamination, the P1 configuration was applied first in this research, and configurations P1 to P4 were executed sequentially. Each process parameter configuration was executed for 3 min, and the total amount of working liquid was approximately 3.6 L. However, some water vaporized in the combustion process; therefore, for each configuration, the collected amount of NCBNFs was slightly lower than 3.6 L.

The manufacturing steps of NCBNFs are detailed as follows. First, the O_2_–C_2_H_2_ torch was ignited, and the proportion of O_2_–C_2_H_2_ was set at the appropriate value (P1–P4). Next, the O_2_–C_2_H_2_ torch was connected to the synthesizer through the burning port, the control valve was opened, and the filtered water was controlled at a flow rate of 1.2 LPM. The smoke was mixed, cooled, and condensed by water mist at this time. The mixture of smoke and water (NCBNFs) flowed into the sample collector, which had an electromagnetic stirrer (PC420D, Corning, USA) configured to stir the NCBNFs continuously at 450 rpm; this maintained favorable suspension and dispersion of the NCBNFs.

Finally, to improve the suspension and dispersion performance of the NCBMs in the base fluid (water), the collected NCBNFs were stirred using a stirrer/hot plate (PC420D, Corning, USA) operating at 450 rpm for 30 min, homogenized at 6000 rpm for 20 min by a homogenizer (YOM300D, Yotec, Taiwan), bathed in an ultrasonic bath (5510R-DTH, Branson, USA) for 30 min, and subjected to intermittent oscillation (25 % amplitude, on/off duty was 10/30 s) by using an ultrasonic liquid processor (Q700, Qsonica, USA) for 20 min. Using these dispersal devices three times effectively prevented a temperature increase in the dispersion equipment and the NCBNFs, achieving favorable dispersion and suspension performance for the NCBNFs in a short period. The dispersed NCBNFs were produced; they were subjected to a series of examinations to determine their characteristics.

## Detection of Characteristics and Analysis of NCBNFs

### Morphology, Crystallization, and Structure Analysis

The morphologies of the NCBMs in the NCBNFs were analyzed using a transmission electron microscope (TEM, H-7100, Hitachi, Japan). The shapes and sizes of the NCBMs were determined. The crystallization of the NCBMs was analyzed using an X-ray diffractometer (XRD, D8 Advanced, Bruker, Germany) with Cu Kα radiation. All peaks measured using XRD were assigned by comparing them with those in the Joint Committee on Powder Diffraction Standards (JCPDS) [26]. Raman spectroscopy (532.15 nm, NRS 4100, Jasco, Japan) was used to detect the Raman shift of the D peaks and G peaks of the NCBMs. The NCBNFs were dropped on glass sheets (20 mm × 20 mm × 0.6 mm) and dried to form carbon films, to be used as test samples for XRD and Raman spectroscopy measurements.

### Production Rate Analysis

The production rate of NCBMs for each process parameter configuration was measured to determine the concentration of NCBMs in the NCBNFs. Because the weights of NCBMs contained in the original NCBNFs were low, each of the four types of NCBNFs were heated in an oven and concentrated to approximately one fourth of its original weight to increase the concentration of each NCBNF. Dried and concentrated NCBMs can be weighed with greater accuracy. For each of the four types of NCBNFs, a 30-g sample was heated using a moisture analyzer (MX-50, A&D, Japan) to remove moisture; the NCBM concentration in the NCBNF sample was then estimated based on the remaining weight (weighing method). Because the highest resolution of the moisture analyzer was 1.0 mg, to improve accuracy, we used a high-precision electronic balance (0.01 mg/42 g, GR202, A&D, Japan) to weigh samples before and after drying. The entire weighing procedure was repeated five times, producing 10 data (each iteration of the procedure produced one datum measured using the moisture analyzer and another datum measured according to the high-precision electronic balance). The five most concentrated data were then averaged as the containing weight of the NCBM. Finally, the weight concentration of each NCBNF was obtained from the containing weight of the NCBMs, the weight of the test sample of the NCBNF, and the concentration ratio of the NCBNF in the oven.

### Optical Characteristics and Suspension Performance Analysis

A UV/VIS/NIR spectrometer (V670, Jasco, Japan) was used to measure the transmittance and absorbance of each NCBNF at wavelengths from 300 to 1200 nm for identifying its optical characteristics. These optical characteristics are helpful for determining the possible applications of the NCBNFs.

To determine the suspension performance of the NCBNFs, they were tested using a static position method, a UV/VIS/NIR spectrometer (V670, Jasco; photometric accuracy ±0.3 % T), and a particle size/zeta potential analyzer (SZ-100, HORIBA, Japan). For the static position method, the NCBNFs were placed into transparent test tubes, and the sediment differences were observed with the naked eye after 24 h. UV/VIS/NIR spectrometry combined with static positioning (2.5-mL NCBNF in a transparent cuvette) was performed to measure the difference in the absorbance of the initial (Abs_*i*_) and static position of each NCBNF after 8 h (Abs_*t*_). The results were used to determine the suspension performance, which was calculated using Eq. (1). A greater absorbance difference ratio (*R*_Abs_) represents more pronounced sedimentation in the NCBNF after 8 h, and conversely, a lower ratio represents higher suspension performance for the NCBNF.

1 $$ {R}_{\mathrm{Abs}}=\left[\left({\mathrm{Abs}}_i-{\mathrm{Abs}}_t\right)/{\mathrm{Abs}}_i\right]\times 100\;\% $$

Particle size analysis and zeta potential analysis are based on the dynamic light-scattering method (DLS), which can be used for simultaneously measuring the particle size and zeta potential of the NCBMs dispersed in a base fluid with a zeta cell to determine the average particle size, particle size distribution, and suspension performance.

### Measurement of Other Fundamental Characteristics

The rheological properties of the NCBNFs were determined using a rheometer (DV3TLVCP, Brookfield, USA; accuracy ±1.0 %) in a cone and plate configuration (cone spindle: CPA-40Z), and the sample temperature was controlled at 25 °C by using an isothermal unit (HW401L, HILES, Taiwan; accuracy ±0.5 °C). The rheological properties of the NCBNFs were tested using the rheometer both with various shear rates (112.5–450.0 s^−1^/15–60 rpm) and at a constant shear rate (262.5 s^−1^/35 rpm for 260 s). The flow state of the samples in the rheometer maintained a laminar flow for rheological measurement procedure.

The specific heat of the test samples was measured using a differential scanning calorimeter (DSC, Q20, TA, USA) with a mechanical refrigeration system (RCS40, TA, USA) in a high-purity nitrogen (5 N) atmosphere. The temperature and calorimetric accuracies of the DSC were ±0.1 °C and ±1.0 %, respectively. The specific heat test method is a standard reference approach, and the standard reference was pure water [27]. To obtain the heat flow data in a temperature range of 20–40 °C, the experimental temperature range covered 10–60 °C, and the heating rate was set at 10 °C/min. The specific heat was calculated using the heat flow data and DSC software (Universal Analysis 2000, TA, USA). To reduce measurement deviations, experiments for determining the specific heat and rheological properties were conducted three times for each NCBNF. The measured data were averaged to obtain the specific heat and rheological properties of the NCBNF.

The thermal conductivity, density, pH, and electrical conductivity of the NCBNFs were measured using a thermal property analyzer (KD-2 Pro, Decagon Devices, USA) with an accuracy of ±5.0 %, a liquid density meter (DA-130N, KEM, Japan) with an accuracy of ±0.001 g/mL, and a pH/conductivity meter (sens ION+ MM374, Hack, USA) with an accuracy of ±0.1 pH and ±0.5 %, respectively, in an isothermal unit (P-20, YSC, Taiwan; accuracy ±0.5 °C) at 25 °C. The experiments were repeated 10 times, and the six closest values were averaged as the test value to reduce experimental deviation.

### Data and Uncertainty Analysis

The experimental results can be presented as a change ratio (*R*) to show the differences in the experimental results of water and NCBNFs; *R* can be expressed as

2 $$ R=\left[\left({D}_{\mathrm{NCBNFs}}-{D}_w\right)/{D}_w\right]\times 100\;\% $$

Uncertainty analysis entailed calculating deviations in the measurements. The uncertainty range of fundamental characteristics of the test samples, such as thermal conductivity (*k*), density (*ρ*), pH, electrical conductivity (*E*), specific heat (*c*_*p*_), and viscosity (*μ*), refer to deviations from the relevant measuring instruments and sample temperature controller. According to standard uncertainty analysis [18], the maximum range of uncertainties in *k*, *ρ*, pH, *E*, *c*_*p*_, and *μ* are within ±5.39, ±2.00, ±2.41, ±2.06, ±1.08, and ±2.24 %, respectively.

## Results and Discussion

**Fig. 2:**
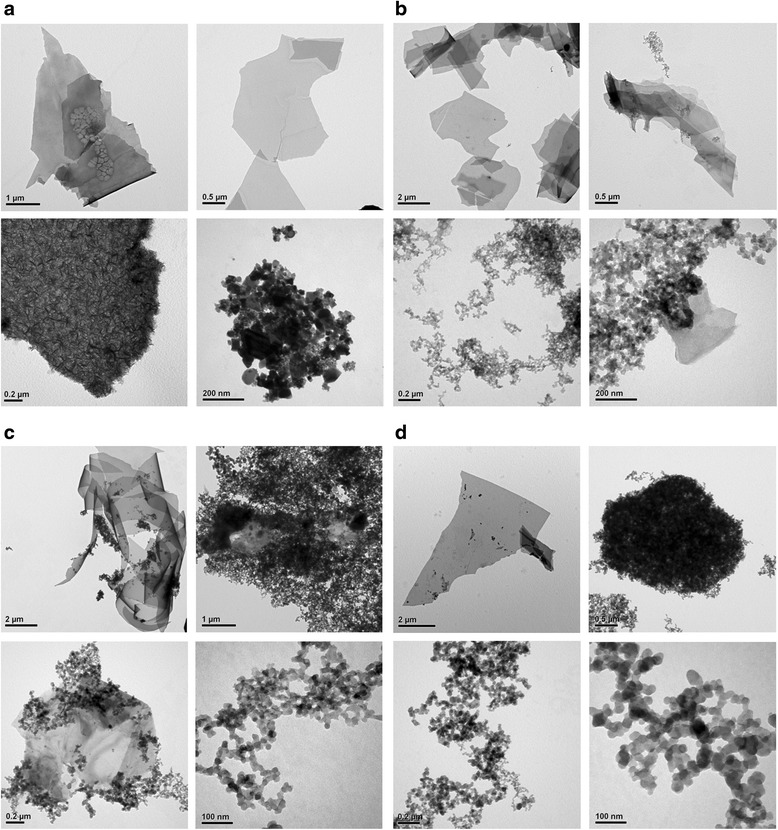
TEM images of NCBMs for process parameters: **a** P1, **b** P2, **c** P3, and **d** P4

Figure 2 shows the TEM image of NCBMs for process parameter configurations P1–P4. As displayed in these TEM images, high proportions of O_2_ in the OAFSS tend to produce flakier NCBMs, and low proportions of O_2_ in the OAFSS tend to produce more spherical NCBMs (of which the diameters are approximately 20–30 nm). However, TEM images show only the local morphology and particle size of each sample; thus, the follow-up to this study will involve using a particle size/zeta potential analyzer to confirm the average particle sizes of suspended NCBMs. In addition, the follow-up will entail subjecting these flaky materials to XRD and Raman spectroscopy to determine whether they are graphene series (graphene, graphene oxide (GO), reduced graphene oxide (RGO)) or nanocarbon (NC).

**Fig. 3:**
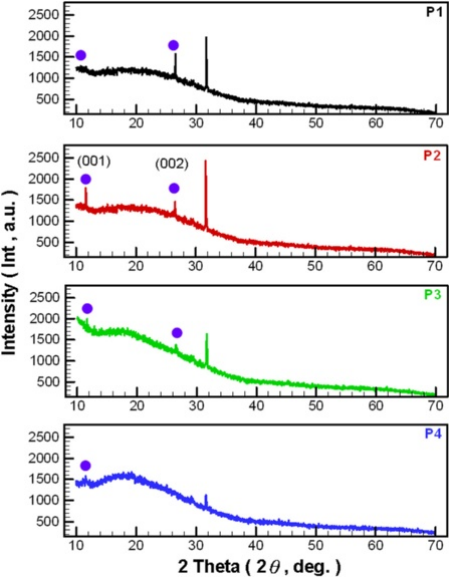
XRD patterns of NCBMs for various process parameter configurations

Figure 3 displays the XRD patterns of NCBMs for process parameter configurations P1–P4. As shown with the XRD patterns, the (0 0 1) diffraction peak was located at 2*θ* ⋍ 11.6°, and from the regional uplift at 15° to 20° for P4 (proportion of O_2_ at the minimum in OAFSS), most of the NCBMs belonged to GO [28]. As the proportion of O_2_ (P4 to P1) increased, the (0 0 2) diffraction peak at 2*θ* ⋍ 26.5° gradually increased, and the composition of the NCBMs gradually had less GO and more RGO [29, 30]. This phenomenon is attributed mainly to the flame temperature, which was lower than 800 °C when the C_2_H_2_ gas was combusted in an atmosphere without additional supply of O_2_ (P4). The configurations that supplied additional O_2_ also raised the flame temperature. The NCBMs generated by higher temperatures produced different degrees of thermal reduction with the water mist, causing the hydroxyl, epoxy, and carboxyl groups in the internal layers of the GO to gradually disappear; thus, GO was gradually reduced to RGO [29, 30]. Studies have disclosed the relatively broad range of XRD diffraction peaks for GO and RGO. Figure 3 shows that these peaks differ significantly, with a sharp prominence at 2*θ* ⋍ 11.6° and 26.5° [30]. Sharply prominent diffraction peaks at 26.5° represent NCBMs that still contain hexagonal graphite-2H (PDF # 897213) [26]. The JCPDS data show only two characteristic peaks of carbon materials. The NCBMs in the present study should also contain amorphous carbon (AC) because their XRD diffraction patterns were assigned after comparing the JCPDS peaks with the overall morphology. The NCBMs produced by different process parameter configurations contained different proportions of GO, RGO, graphite-2H, and AC; these differences are discernible in the measurement results of TEM and XRD.

**Fig. 4:**
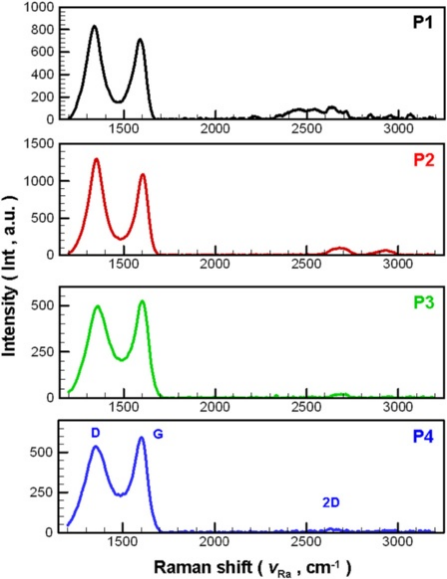
Raman spectra of NCBMs for various process parameter configurations

Figure 4 shows the Raman spectra of NCBMs produced under configurations P1–P4; the figure shows only the Raman spectra of one test point, although five test points were available on the test sample. Table 1 shows the averaged data of Raman spectra, originally measured at five points per sample, for NCBMs produced through configurations P1–P4. The D and G peaks are predominant features in the Raman spectra located at approximately 1324–1346 and 1490–1691 cm^−1^, respectively [30–32]. Only pristine graphite typically presents a prominent G peak at 1584.5 cm^−1^ to correspond to the first-order scattering of E_2g_ mode in the Brillouin zone, thus indicating that the structure of graphite is highly regular [32, 33]. In the Raman spectra of GO, the G peak is broadened, and the D peak becomes prominent; this indicates that the C=C double bonds in the graphite layers were destroyed after oxidation and numerous carbon atoms changed from sp^2^- to sp^3^-hybridized carbon. The intensity of *I*_D_/*I*_G_ (intensity of the D peak/intensity of the G peak) represents the ratio of sp^3^-hybridized carbon and sp^2^-hybridized carbon; in RGO, decreased values of *I*_D_/*I*_G_ indicate increased hybridized sp^2^ and decreased hybridized sp^3^. Theoretically, *I*_D_/*I*_G_ should decline when GO is reduced to RGO, and a lower *I*_D_/*I*_G_ shows a more complete reduction of GO to RGO [30, 34].

**Table 1:** List of Raman spectroscopy for NCBNFs from various process parameter configurations

Items		Process parameter no.			
		P1	P2	P3	P4
*I*_D_	cm^−1^	1338.08	1353.11	1355.52	1355.95
	Int.	526.92	933.81	291.33	465.35
*I*_G_	cm^−1^	1590.88	1607.11	1600.92	1601.75
	Int.	445.03	804.99	308.73	512.87
*I*_2D_	cm^−1^	2638.68	2681.11	2675.92	2668.15
	Int.	78.03	52.27	16.14	16.03
*I*_D_/*I*_G_		1.20	1.16	0.96	0.91
2*I*_D_/*I*_G_		0.16	0.06	0.07	0.03

As shown in Fig. 4, the intensity of *I*_D_/*I*_G_ increases with the proportion of O_2_; an increasing proportion of O_2_ raises the flame temperature. The reduction of GO should lessen the intensity of *I*_D_/*I*_G_ [30, 34]. That increasing the proportion of O_2_ tends to promote the reduction of GO to RGO is unexpected. This phenomenon resembles results for the reduction of GO to RGO by using the chemical reduction method [35–38]. Stankovich et al. [35] found that reduction increases the number of small aromatic domains in RGO, which leads to an increase in the *I*_D_/*I*_G_ ratio. However, by simultaneously considering the analytical results of the Raman spectra and XRD patterns, one can deduce that GO should be gradually converted into RGO as the configuration changes from P4 to P1. The reducing flame temperature increases with the O_2_ supply; the oxygen atoms cannot be removed effectively by forming a double bond in the GO layers; thus, *I*_D_/*I*_G_ increases. The summarized data of Raman spectra listed in Table 1 show that the OAFSS can produce NCBMs containing more GO when the O_2_ flow rate is lower than 1.0 LPM and NCBMs containing more RGO when the O_2_ flow rate is higher than 1.0 LPM. In addition, the *I*_2D_/*I*_G_ of the Raman spectra for each test sample was low, showing that multilayer stacks and agglomeration existed for each test sample. The NCBMs produced by OAFSS with different process parameter configurations contained different proportions of GO, RGO, graphite-2H, and AC. However, the present study could not determine the proportion of each sample element; hence, quantitative analysis requires further study in this regard.

**Fig. 5:**
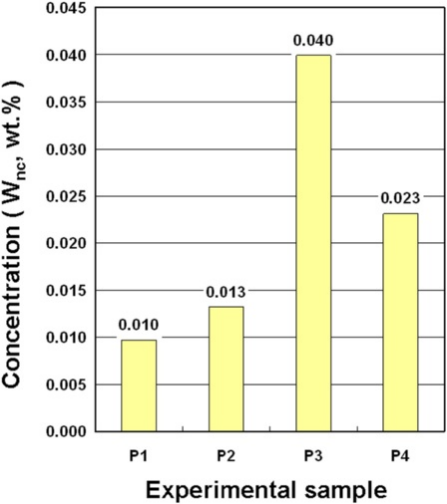
Concentrations of NCBNFs for various process parameter configurations

Figure 5 shows the concentrations of the NCBMs in NCBNFs for configurations P1–P4, measured using the weighing method. The P1, P2, P3, and P4 configurations produced NCBM concentrations of 0.010, 0.013, 0.040, and 0.023 wt%, respectively, in the NCBNFs. The results revealed that aside from P4, the production rate (concentration) of NCBMs and the O_2_ flow rate presented an inversely proportional trend. In addition, P4 produced the greatest amount of black smoke in the preparation process, and should have exhibited the highest production rate of NCBMs, but P4 produced dark fumes that exited the exhaust pipe, and the water mist did not capture the chemicals that darkened those fumes. Therefore, the production rate of P4 was lower than that of P3 because the water mist was inefficient. The P4 process produced smoke by C_2_H_2_ combustion with O_2_; the smoke then rose sharply in the synthesizer, failed to mix with the mist, and was lost. The collection efficiency of the OAFSS can be improved by increasing the water flow rate, spray range, and spray form.

**Fig. 6:**
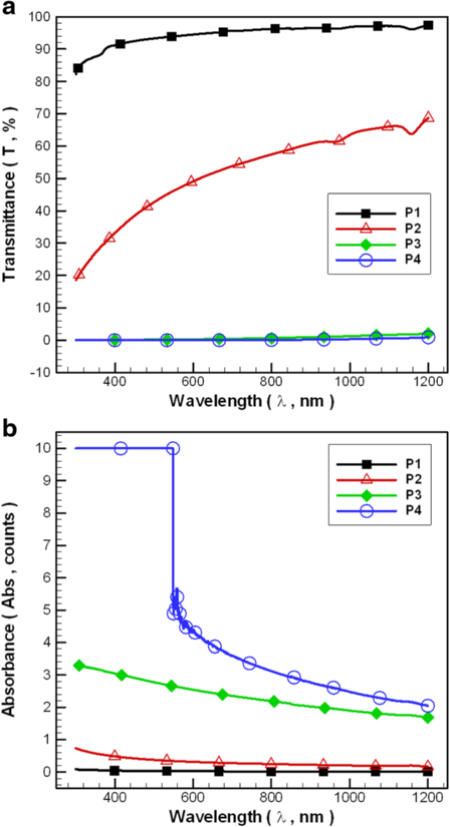
Transmittance and absorbance of NCBNFs for various process parameter configurations: **a** transmittance and **b** absorbance

Figure 6 shows the transmittance and absorbance curves of the NCBNFs for configurations P1–P4. For every sample, the transmittance and absorbance curves obtained at different wavelengths exhibited similar wavelength trends higher than 600 nm but with different values. The NCBNF of P4 was nearly impenetrable (the highest spectrometer absorbance is 10) at wavelengths lower than 600 nm; therefore, the transmittance and absorbance curves of different NCBNFs are unsuitable for a comparison lower than 600 nm. Low O_2_ flow rates in the OAFSS can produce high concentrations of NCBNFs according to the change of the transmittance and absorbance of the NCBNFs. However, the concentration results from spectral analysis and the weighting method differed. This discrepancy occurred because different C_2_H_2_ and O_2_ ratios may produce different materials or particle sizes; the produced NCBMs thus have distinct optical characteristics. Therefore, this study used the weighing method to determine quantitative concentrations.

**Fig. 7:**
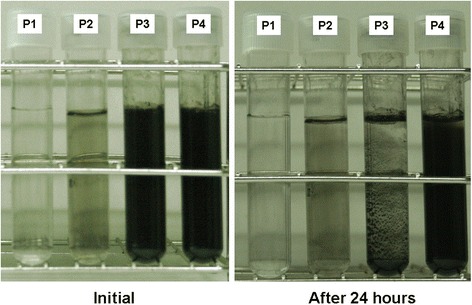
Photograph of experimental results obtained using the static position method

Figure 7 displays a photograph of the experimental results obtained using the static position method for the products of configurations P1–P4 after 24 h of static positioning. As shown in the figure, P3 had the worst suspension performance, followed by P4, P2, and P1. However, P1 and P2 had low concentrations; the naked eye cannot discern any differences before and after static positioning; therefore, the spectrometer had to be used to confirm the difference in suspension performance between P1 and P2.

**Fig. 8:**
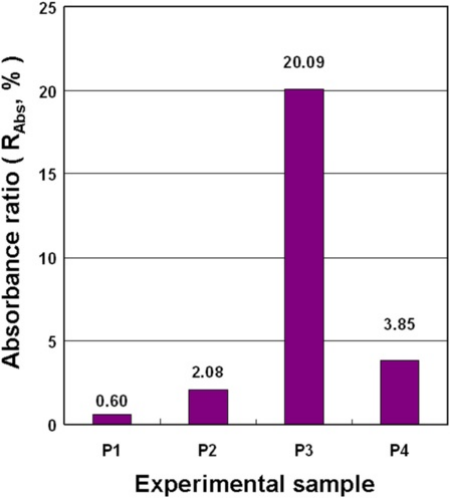
Absorbance difference ratios for the static position method applied to the NCBNFs from various process parameter configurations

Figure 8 displays the *R*_Abs_ of the NCBNFs produced by P1–P4 at a wavelength of 800 nm. The samples were statically positioned for 8 h to analyze the suspension performance of the NCBNFs by using Eq. (1). High *R*_Abs_ represents more severe sedimentation; conversely, low *R*_Abs_ represents greater suspension. As shown in the figure, the suspension performance test results in Fig. 7 exhibited the same trend as that of the *R*_Abs_ results. After 24 h of the static position method, the naked eye could see (Fig. 7) that P3 had the worst suspension performance, followed by P4, P2, and P1. However, the concentration of P3 was the highest for all the samples; thus, P3 had the most suspended NCBMs, and the highest probability of particle agglomeration, which caused the poor suspension performance of P3. The *R*_Abs_ was compared to the concentration of each sample; that comparison demonstrated the inverse relationship between suspension performance and concentration.

**Fig. 9:**
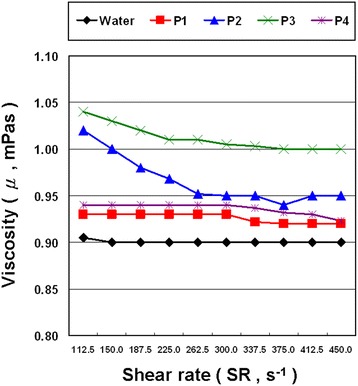
Viscosity of NCBNFs for various shear rates and process parameter configurations

The viscosities of water and NCBNFs produced from configurations P1–P4 are plotted as a function of the shear rate (112.5–450 s^−1^) for a sample temperature of 25 °C in Fig. 9. As shown in the figure, the viscosity of water remained almost constant with an increase in the shear rate, indicating Newtonian behavior, and the viscosity of all the NCBNFs decreased with an increase in the shear rate, indicating non-Newtonian and shear-thinning behavior. This phenomenon is mainly due to the individual aggregation of NCBMs in NCBNFs; aggregations of NCBMs in NCBNFs start to break apart, and NCBMs align in the direction of the shear flow as the shear rate increases; consequently, the viscosity decreases. Therefore, NCBNFs exhibit shear-thinning behavior. The viscosities of P2 and P3 markedly decrease with the shear rate before 262.5 s^−1^, and those of P1 and P4 remain unchanged. This phenomenon is mainly due to the size and structure of aggregation of NCBMs in NCBNFs. Great aggregations of NCBMs or aggregations of NCBMs with looser structure in NCBNFs are likely broken apart, thus causing significantly decreased viscosity with increasing shear rates.

The higher concentration of NCBMs typically shows greater viscosity and more intense shear-thinning characteristics. When the concentrations of NCBMs in the NCBNFs increased, the NCBNFs became increasingly more disordered as more aggregates formed, and the viscosity rose [39, 40]. Furthermore, the manner in which NCBNFs interact with water, particle size, and NCBM morphology also affects the viscosity of NCBNFs. Therefore, although the concentration of P4 was higher than that of P2, the viscosity of P4 was lower than that of P2, and P4 exhibited less shear-thinning behavior.

**Fig. 10:**
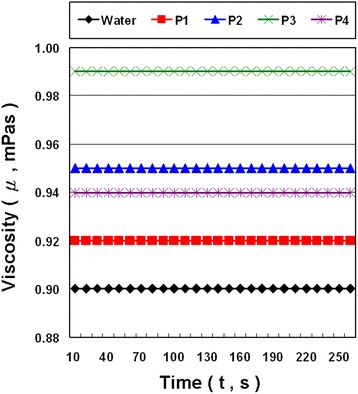
Viscosity of NCBNFs at shear rates of 262.5 s^−1^ for various process parameter configurations

The viscosities of water and NCBNFs produced by P1–P4 are plotted as a function of the test time for a sample temperature of 25 °C at a specific shear rate (262.5 s^−1^) in Fig. 10. The viscosity of each NCBNF exhibited a constant value at a shear rate of 262.5 s^−1^. The viscosity of P4 was lower than that of P2 because of differences in interface characteristics, particle size, and NCBM morphology. TEM images show that the NCBMs of P4 were mostly spherical NCBMs of approximately 30 nm and few P4 NCBMs were flaky. However, P2 had more flaky NCBMs and fewer spherical NCBMs. Because NCBMs of a uniform size have less friction, and because of the flow resistance between NCBMs and NCBMs or between NCBMs and water molecules, the viscosity of P4 was lower than that of P2 despite the concentration of P4 being higher than that of P2. The viscosities of P1, P2, P3, and P4 were higher than that of water by 2.22, 5.56, 10.00, and 4.44 %, respectively.

Table 2 lists the experimental results of average particle size, zeta potential, thermal conductivity, density, pH, electrical conductivity, specific heat, and viscosity of NCBNFs produced with various process parameter configurations. The average NCBM particle size was measured 10 times, and the five closest test data were averaged as the test result. The experimental results revealed that the average NCBM particle size for P3 was up to 793 nm, which may correspond to the suspension performance of P3 (Figs. 7 and 8). This may confirm that P3 had the worst NCBM suspension performance. Furthermore, the probability of aggregation can be relatively high for a high concentration of NCBMs in an NCBNF. This can result in a large NCBM particle in the suspension. P1 yielded the smallest average particle, followed by P4, P2, and P3. However, after many rounds of particle size measurements, the particle size distribution appeared to be bimodal. A higher proportion of oxygen in the OAFSS corresponded with a higher probability for bimodal distribution. This phenomenon was consistent with the TEM test results. High amounts of oxygen produced numerous flaky NCBMs. When these flaky NCBMs were measured using the DLS method, the results revealed that the particles were large and that the particle size distribution was bimodal. Highly concentrated NCBNFs produced using configurations P3 and P4 exhibited less bimodality. The distribution peaks for small and large particles were mainly due to spherical particles and flaky NCBMs, respectively.

**Table 2:** Results of other fundamental characteristics for NCBNFs from various process parameter configurations

	Experimental data					*R* (%)			
	Water	P1	P2	P3	P4	P1	P2	P3	P4
Average particle size (nm)	–	207.68	380.52	792.82	255.34	–			
Zeta potential (mV)	–	−22.96	−24.18	−18.32	−26.36				
Thermal conductivity (W/m °C)	0.602	0.606	0.622	0.624	0.631	0.68	3.34	3.71	4.85
Density (kg/m^3^)	997.95	998.09	998.14	998.29	998.26	0.01	0.02	0.03	0.03
pH	7.68	7.64	7.47	7.59	7.47	−0.55	−2.65	−1.11	−2.65
Electrical conductivity (μS/cm)	113.40	148.31	122.14	118.90	114.06	30.79	7.71	4.85	0.58
Specific heat (kJ/kg °C)	4.18	4.17	4.13	4.10	4.10	−0.32	−1.08	−1.91	−1.89
Viscosity (mPa s)	0.90	0.92	0.95	0.99	0.94	2.22	5.56	10.00	4.44

The zeta potential (*V*_z_) values that are far from zero (high absolute value) indicate NCBNFs with excellent suspension performance. The highest *V*_z_ was P4, followed by P2, P1, and P3, as shown in Table 2. P3 produced the largest average particle, the lowest *V*_z_, and the worst suspension performance, a result that was consistent with that obtained using the static position method. However, the *V*_z_ values of other samples (P1, P2, and P4) for suspension performance had certain differences with the results obtained using the static position method. This phenomenon was mainly due to different test methods and the measurement deviation. The *V*_z_ value of an NF is typically within the range of ±10 to ±30 mV, which means that the NF exhibits incipient instability. If the *V*_z_ value of an NF is within the range of ±30 to ±40 mV, this means that the NF exhibits moderate stability. If the *V*_z_ value of an NF is within the range of ±40 to ±60 mV, this means that the NF exhibits favorable stability. When the *V*_z_ of an NF is greater than ±60 mV, that NF exhibits excellent stability [41, 42]. The *V*_z_ values of NCBNFs with various process parameters of the OAFSS were within the range of −18 to −26 mV; thus, the OAFSS products exhibited incipient instability. Therefore, the suspension performance of these NCBNFs must be appropriately augmented with a dispersant, surfactant, or agent to adjust the pH value. Such augmentation can improve the suspension performance when NCBNFs are used in heat exchange systems with long-term stability.

The thermal conductivity test results for these NCBNFs revealed that P4 had the highest thermal conductivity, followed by P3, P2, and P1, as shown in Table 2. The enhancement rates of thermal conductivity for P1, P2, P3, and P4 were respectively 0.68, 3.34, 3.71, and 4.85 % higher than that of water. Increases to the concentrations of nanoparticles in an NF generally raise the thermal conductivity of that nanofluid. However, the material, average particle size, and NCBM morphology within these four samples (P1–P4) were dissimilar; therefore, the enhancement rate of the thermal conductivity did not necessarily increase with a rising concentration of NCBMs. The NCBM concentration of P4 was approximately 2.3 times that of P1; the enhancement rate of the thermal conductivity for P4 was approximately 7.1 times that of P1. The NCBM concentration of P4 was approximately 1.8 times that of P2; the thermal conductivity enhancement rate of P4 was approximately 1.5 times that of P2. The NCBM concentration of P4 was roughly 0.6 times that of P3; the thermal conductivity enhancement rate of P4 was approximately 1.3 times that of P3. P2 is the best option if one desires to optimize the NCBM concentration and the thermal conductivity enhancement rate. However, P4 is the best option for optimizing the process time, fuel cost, and thermal conductivity enhancement rate. Although no sample had a high thermal conductivity enhancement rate, all these NCBNFs had extremely low concentrations of NCBMs; thus, their performance levels were remarkable.

The test results of density for NCBNFs revealed that the density increased with the NCBM concentration. The difference in density for P1–P4 was negligible within the range of instrument deviation because the NCBM concentration was low. The enhancement rates of density for P1, P2, P3, and P4 were respectively 0.01, 0.02, 0.03, and 0.03 % higher than that of water.

The pH test results for NCBNFs revealed that the pH values were lower than that of water because during the combustion process, CO_2_ dissolved in water to form carbonic acid, which slightly lowered the pH of the NCBNFs. Differences in pH were minor among the fluids produced through P1–P4, but the range of pH was within 7.5 ± 0.15. Therefore, the pH values of P1–P4 did not differ significantly. The decline rates of pH for P1, P2, P3, and P4 were respectively 0.55, 2.65, 1.11, and 2.65 % higher than that of water.

A high concentration of solid particles in an NF typically increases the electrical conductivity of that NF, but different NCBNFs have different NCBMs. In this study, the enhancement rate of electrical conductivity did not increase with the concentration of NCBMs. However, a relationship can be found between the trend of electrical conductivity and the XRD and Raman test results. If the O_2_ ratio in the O_2_–C_2_H_2_ flame is high, most NCBMs are RGO and crystalline graphite. If the O_2_ ratio in the O_2_–C_2_H_2_ flame is low, most NCBMs are GO and AC. The electrical conductivity of RGO and crystalline graphite is high, whereas that of GO and AC is poor; therefore, the electrical conductivity of each NCBNF is relatively different. Furthermore, the NCBM concentration, particle size, and suspension performance all affect the electrical conductivity of an NCBNF. The enhancement rates of electrical conductivity for P1, P2, P3, and P4 were respectively 30.79, 7.71, 4.85, and 0.58 % lower than that of water.

The specific heat values of NCBMs were substantially lower than that of water. Therefore, the specific heat values of NCBNFs decrease with increasing concentrations of NCBMs. The differences in specific heat for P1–P4 were negligible because the NCBM concentrations were low. The decline rates of specific heat for P1, P2, P3, and P4 were respectively 0.32, 1.08, 1.91, and 1.89 % higher than that of water. The viscosities listed in Table 2 are the average values of the test results shown in Fig. 10.

## Conclusions

In this study, an OAFSS was used to fabricate NCBNFs in a one-step synthesis process. The NCBNFs were manufactured through O_2_–C_2_H_2_ combustion at different flow rate ratios of O_2_/C_2_H_2_ (P1–P4) and a constant flow rate of cooling water. The characteristics of the NCBNFs and suspended NCBMs were examined using suitable instruments and test methods. The findings of this study are summarized as follows:

1. The NCBM morphologies of the NCBNFs were mainly flaky and spherical, and the diameters of the spherical NCBMs measured approximately 20–30 nm.
2. The NCBMs that contained GO and AC resulted from O_2_ flow rates lower than 1.0 LPM, and the NCBMs that contained RGO, graphite-2H, and AC resulted from O_2_ flow rates higher than 1.0 LPM in this study.
3. The process parameter configurations of P1, P2, P3, and P4 produced NCBM concentrations of 0.010, 0.013, 0.040, and 0.023 wt%, respectively, in the NCBNFs.
4. The rheological properties of all the NCBNFs exhibited non-Newtonian and shear-thinning behavior.
5. The enhancement rates of thermal conductivity for P1, P2, P3, and P4 were respectively 0.68, 3.34, 3.71, and 4.85 % higher than that of water. The thermal conductivity enhancement rates for these NCBNFs should be deemed excellent, considering the extremely low concentrations of NCBMs in these NCBNFs.
